# Two kinds of embryo research: four case examples

**DOI:** 10.1136/medethics-2021-108038

**Published:** 2022-05-09

**Authors:** Julian Savulescu, Markus Labude, Capucine Barcellona, Zhongwei Huang, Michael Karl Leverentz, Vicki Xafis, Tamra Lysaght

**Affiliations:** 1 Faculty of Philosophy, University of Oxford, Oxford, UK; 2 Biomedical Research Group, Murdoch Childrens Research Institute, Parkville, Victoria, Australia; 3 Centre for Biomedical Ethics, Yong Loo Lin School of Medicine, National University of Singapore, Singapore; 4 Saw Swee Hock School of Public Health, National University of Singapore, Singapore; 5 NUS Bia-Echo Asia Centre for Reproductive Longevity and Equality, Singapore; 6 Institute of Molecular and Cell Biology, Agency of Science, Technology and Research, Singapore; 7 Obstetrics and Gynaecology, National University of Singapore, Singapore; 8 Mechanobiology Institute, National University of Singapore, Singapore

**Keywords:** Embryo Research, Embryos and Fetuses, Ethics, Ethics Committees

## Abstract

There are ethical obligations to conduct research that contributes to generalisable knowledge and improves reproductive health, and this should include embryo research in jurisdictions where it is permitted. Often, the controversial nature of embryo research can alarm ethics committee members, which can unnecessarily delay important research that can potentially improve fertility for patients and society. Such delay is ethically unjustified. Moreover, countries such as the UK, Australia and Singapore have legislation which unnecessarily captures low-risk research, such as observational research, in an often cumbersome and protracted review process. Such countries should revise such legislation to better facilitate low-risk embryo research.

We introduce a philosophical distinction to help decision-makers more efficiently identify higher risk embryo research from that which presents no more risks to persons than other types of tissue research. That distinction is between future person embryo research and non-future person embryo research. We apply this distinction to four examples of embryo research that might be presented to ethics committees.

Embryo research is most controversial and deserving of detailed scrutiny when it potentially affects a future person. Where it does not, it should generally require less ethical scrutiny. We explore a variety of ways in which research can affect a future person, including by deriving information about that person, and manipulating eggs or sperm before an embryo is created.

## The ethical importance of research

We conduct research to systematically examine and gain insight into the complexities of life and the world around us, and use this knowledge to improve the human condition. Research for such purposes that is conducted in accordance with international standards is therefore an ethically good enterprise.^1^ Given our ability to systematically examine aspects of human existence that deeply impact our lives, research aimed at promoting human health and well-being is a moral imperative. This imperative also applies to research that has the potential to improve fertility and reproductive health. If we accept that procreation is a moral good, then society has an obligation to support scientific research that is broadly aimed at improving fertility.

This obligation arises from two considerations: first, improving fertility for those segments of the population requiring scientific interventions to procreate enables such individuals to pursue a life that is meaningful to them while at the same time not infringing on others’ fundamental interests^2^ and second, such scientific research addresses issues relating to the right to fair and reasonable access to healthcare. If we are to consider the instrumental value of procreation, the motivation becomes stronger in countries where major demographic shifts are contributing to ageing populations with decreasing fertility rates that have fallen well below replacement levels.^3 4^ For example, total fertility rates in 2018 for the USA were 1.7, 1.7 for the UK, 1.4 for Japan and only 1.1 for the Southeast Asian city-state of Singapore.^5^ With an estimated replacement fertility rate of 2.1,^6–8^ countries such as these must rely on immigration policies to maintain their current population levels.

We acknowledge that the effects of unchecked population growth in combination with ever-increasing consumption rates impact on environmental degradation and the critical preservation of limited resources.^9^ However, we do not view the solution to population growth control as being achieved by depriving only some persons from procreating, simply because they face fertility issues. Rather, there are global collective moral obligations that need to be considered in reducing consumption and human impacts on environmental health and climate change, if we are to discuss this complex and confronting problem with issues of justice in mind. In this paper, we are not able to further elaborate on this important aspect of the argument.

While both men and women are living longer in these countries, they are also deciding to have children later, which affects the health and quality of their reproductive tissues.^3 10 11^ Maternal age is one of the strongest predictors of oocyte quality.^12^ Therefore, the quality and quantity of ovarian follicles are vital for a woman’s reproductive lifespan. A condition known as premature ovarian insufficiency (POI), where the loss of normal ovarian function is due to the loss of ovarian follicles (ie, loss of quantity) before the age of 40 years, affects approximately 1% of women under 40 years old and 0.1% of women under 30 years of age.^13^ Hence, this exemplifies that quantity is equally important in a woman’s reproductive lifespan, especially when POI occurs in young women, and ovarian follicles and the oocytes are perceived to be of better quality than in older women. Furthermore, young women who have undergone gonadotoxic treatment and have high numbers of good quality ovarian follicles destroyed can have problems conceiving due to the sheer low number of ovarian follicles and will go into the POI state and be rendered infertile despite the likely presence of low numbers of perceived better-quality oocytes due to her younger age (barring the fact that these oocytes could have been affected by the medical treatment).

During normal ageing in women, the culmination of clinical menopause occurs because of loss of both quality and quantity of ovarian follicles, which signals the end of a woman’s reproductive lifespan. Thus, both quantity and quality of ovarian follicles are equally vital for a woman’s reproductive lifespan. Research on human reproductive cells and tissues can thus improve the fertility of couples and help them conceive healthy babies with less risk of complications in pregnancy. It can also promote the procreative choices of women who want to delay parenthood until later in life when they are likely to be more financially secure.

While technological fixes alone will not resolve diminishing fertility rates, there are social and moral imperatives to support this research because it benefits not only individual parents, but society as well through improved reproductive health and knowledge of the developmental and ageing processes. Given these imperatives, regulation and ethical review of fertility research ought to be constructive and not unreasonably obstructive. In this paper, we draw a philosophical distinction between high-risk and low-risk research with reproductive tissues, especially that which involves human embryos, that can facilitate constructive regulation and ethical review.

## Human embryo research

Research with human embryos in most countries is highly regulated. Some countries, including Austria and Italy, have prohibited any research that results in the destruction of human embryos.^14 15^ Other countries, including Australia, the UK and Singapore, allow destructive embryo research with appropriate ethical review and under certain conditions. For example, some jurisdictions allow embryo research when limited to supernumerary embryos or when there is an absence of an adequate alternative. Following the prohibition on developing human embryos beyond 14 days of fertilisation, most of these countries also mandate the destruction of embryos after this time period.^15^

There are at least two reasons for this variability. The first is because of different views of the moral status of the embryo. Embryo research often destroys embryos, and some hold the view that embryos have moral status and that it is wrong to destroy them, even as wrong as killing a person.^16 17^ Other people do not hold such views and believe that embryos do not hold moral status, and therefore, in accordance with this view, it is not wrong to destroy them for scientific research that may benefit future patients.^16 18^ However, the most common position is the gradualist view of moral status: that is, the fetus gradually acquires increasing moral status during pregnancy, meaning that abortion at advanced gestations requires much stronger justification. This view holds that human embryos have a limited degree of moral status, meaning that their use in research should be balanced against the benefits and that their use in research is only ethically acceptable if the benefits outweigh the presumed ethical violation of destroying them. This view may be true but our distinction is still important. Even if an early embryo has low value on this view, the future person view states it should be accorded higher moral status if it will, or would likely, become a future person.

Second, if the embryo will not become a future person, but still retains some status or value on this view, it is necessary to compare this status or value to other contexts. In most western liberal countries, abortion and contraception are available on demand. This suggests a view of very low moral status. Indeed, in many jurisdictions, embryos surplus to a couple’s needs must be destroyed by law after a certain period of time, even when they could be donated to childless couples who desire them. This again suggests a view of very low status or value. And even if they did have some value or status, this must be considered against the very real value of well-designed research. To treat embryos in research differently to research in in vitro fertilisation (IVF), or reproduction generally, is a form of ‘research exceptionalism’.

Ethics committees must make decisions when national rules are unclear regarding in vitro cultures of viable (surplus) embryos and embryo model research. The International Society for Stem Cell Research leaves it up to each country to decide on the extension of the 14-day rule. However, our distinctions apply to these situations and the 14-day rule is irrelevant. If an embryo is earlier than 14 days, but will turn into a person, then it should be treated as if it were a person, even though it is not. For example, a modification on a blastocyst which would be implanted should be treated in the same way as a modification of an infant.

Research should be treated on a par with clinical practice and termination of pregnancy. Fourteen days does not signify anything of relevance to the future person. Jeff McMahan argued that an individual organism begins at 14 days, when cells begin to co-ordinate to form an organism, just as when tribes begin to co-operate there emerges a nation.^19^ This argument is dubiously based on claims about the lack of cellular organisation before 14 days. Yet, even if an individual begins to exist at 14 days, rather than being a clump of cells, that individual is not yet a person. According to the future person distinction we have presented, it is implantation (or ectogestation) that matters, not embryonic stage of development. We acknowledge that the issue is complex and we do not attempt to settle the matter here.

Furthermore, in this paper, we only consider jurisdictions that allow fertility treatments through, for example, IVF and the inevitable destruction of embryos that are discarded or surplus for treatment purposes. These jurisdictions have accepted that research with embryos that would otherwise be destroyed is ethically acceptable. In fact, some jurisdictions, such as the UK, even allow the creation of embryos solely for the purposes of research. However, we do not consider this scenario; we focus only on embryos created in clinical contexts for the purposes of fertility treatment.

The second, and more important, reason for the contention surrounding embryo research is that an embryo has the potential to become a future person whose life can be fundamentally changed by what was done at this very vulnerable and profound stage of development. It is essential that anything done to an embryo, or information derived from it, is consistent with the well-being of the future person *and* does not expose that person to unreasonable risks. This requires minimising risk, ensuring that experimentation is based on the best available evidence and reasoning, and ensuring that risks are proportionate to the expected benefits, either to the future person (or to others, such as other fertility patients).^15^ Several layers of evaluation can help to achieve these aims: first evaluation by the researchers themselves, then by an ethics committee, then by the parents or producers of the embryo, who must ultimately give their informed consent for the research.

Various ethical review procedures in different countries instantiate these basic requirements in different forms. Our goal is not to question these requirements directly but question the scope. Despite some countries having relatively permissive legislation in relation to embryo research, such as the UK, many countries (such as Australia^16^ and Singapore) have legislation which imposes onerous requirements on low-risk embryo research, such as observational studies. We will argue it is important ethically to differentiate between two different kinds of embryo research: research on embryos that will foreseeably result in a future person and research on embryos that will not foreseeably result in a future person because of the decision of either the donor/provider or their treating physician not to implant the embryo. Let us call these two kinds of research: Future Person Embryo Research and Non-Future Person Embryo Research.

## The nature of the future person embryo research and non-future person embryo research distinction

Future person embryo research includes anything which is done to an embryo that will be or could be implanted into a woman’s uterus. This might include embryo biopsy, preimplantation genetic screening, whole genome analysis, observational research (including using time lapse imaging), embryo culture, media from embryo culture used for non-invasive testing and gene-editing in embryos. Experimentation on gametes (sperm and eggs) is not embryo research per se but may raise the same ethical issues as future person embryo research if those gametes might be used to create an embryo which is transferred to the woman’s uterus.

Non-future person embryo research involves any procedure done to an embryo that will never be implanted because of the decision of the patient or their treating physician. This category includes embryos that are clinically assessed as being unsuitable or nonviable for transfer, as well as embryos that will not form a future person because of independent decisions not to implant or use them for fertility treatment. For example, the donor’s family is complete and they do not wish to donate them to other couples or dispose of them as biological waste. Experimentation on gametes that will not create an embryo for fertility treatment may also fall into this category. The donors must give their informed consent for use of these tissues for research purposes.

There are thus four relevant categories to embryo research:

1. Research on gametes that are not intended to, or will not foreseeably, create an embryo for treatment.
2. Research on non-viable embryos that will not be used for treatment.
3. Research on viable (surplus) embryos that will not foreseeably be used for implantation.
4. Research on viable embryos or gametes that will be used for treatment.

It is category 4 that we argue raises special ethical issues related to future persons.

## Why does this distinction matter?

This distinction matters because future person embryo research generally requires high-level scrutiny to protect future persons whereas non-future person embryo research, although embryo research, does not require this level of scrutiny, including risk-benefit evaluation because no sentient person will ever be directly affected. An embryo or fetus does not become a person until after birth.^19 20^ While some theologians have argued that personhood begins at fertilisation^21 22^ many others disagree,^23 24^ and in jurisdictions that allow destructive embryo research, preimplantation embryos are not attributed with legal or moral personhood. Even if human embryos are attributed with some special status, which aligns with the prevailing gradualist view of the moral status of human embryos, it is insufficient to protect them from destructive research. It is only when embryos are to be implanted into a biological womb that they can foreseeably turn into a person. At that time, an embryo should be treated as if it were a person, even though it is not, if there is the intention to gestate that pregnancy to term.

For example, an experimental modification made to an embryo which would be implanted should be treated in the same way if that modification were made to an infant. That would be future person research and warrants high levels of ethical scrutiny. However, if that embryo will not ever be implanted to foreseeably become a person, then it is non-future person research and should require much less scrutiny.

In a nutshell, future person embryo research is contentious and warrants detailed ethical review; non-future person embryo research is not as contentious and requires much lower level, faster review (still conforming to the requirements for ordinary human tissue research and legislation). What matters ethically is whether there could be a future person resulting from this embryo and whether the person subsequently borne out of the research could be adversely affected in the future by what is done now.

However, there are exceptions. Future person embryo research can also be relatively uncontentious, and not require high-level research ethics review. For example, in non-interventional observational research, nothing is done to the embryo and nothing interferes with its growth that could affect the life of a future person. Such research may involve the collection of data on the future person. However, with the informed consent of the future person’s prospective parents and appropriate data protection protocols, such research would ordinarily be considered as no-more-than minimal risk. The research would come under the purview of ethics review, but would not warrant high levels of ethical scrutiny. Similarly, non-future person embryo research can occasionally be contentious for reasons other than the mere fact it destroys an embryo, for example, when research on discarded embryos is potentially discriminatory,^17^ frivolous or lacks positive social value.^18^

## Application of this distinction to test cases

Table 1 provides examples of test cases that exemplify the distinctions between the categories of embryo research discussed above so as to clarify the appropriate level of ethical oversight required. These cases raise additional ethical issues which are not the focus here, so we refrain from elaborating on ethical concerns such as privacy, confidentiality, consent and deidentification, particularly in case 1. In case 2, there are also issues relating to incidental findings, such as the discovery of chromosomal imbalances in the embryos, for example, chromosome translocations that may be inheritable from the parents and will have implications for the couple to be investigated to confirm their karyotypes and then decide whether there are repercussions for their future plans for conceiving.

**Table 1:** Case studies highlighting issues in some jurisdictions in human reproduction research

Case study	Biosample of interest	Stakeholders
**Case 1: Time lapse imaging and embryo selection** Time-lapse monitoring of embryos versus conventional assessment of embryo morphology for selection for embryo transfer Principle of study: Non-invasive monitoring of viable embryos developing in a time-lapse machine unperturbed compared to daily conventional assessment of embryo morphology on reproductive outcomes Expected outcomes: Comparison of embryological and clinical data as well as reproductive outcomes from embryos monitored via the time-lapse machine vs conventional morphological assessment using light microscope (done routinely in all IVF labs)	Observing developing embryos continuously using the time lapse machine (already in clinical usage in some countries) No manipulation of embryos—data gathered are just morphological criteria and demographics of women	IVF team—embryologists, physicians Patients—comparison of two methods which are done routinely (conventional assessment) and the continuous time lapse monitoring in a machine that has been approved
**Case 2: Confirmation of diagnosis of aneuploidy** Confirmation of the karyotype of aneuploid blastocysts (day 5 embryos) identified from trophectoderm biopsy. Aneuploid blastocysts have abnormal no of chromosomes and result in miscarriages and reproductive failure Principle of study: Blastocysts tested with preimplantation genetic screening techniques by trophectoderm biopsy to confirm its karyotype by assessing these embryos in totality by disaggregating the embryos—inner cell mass (which gives rise to the embryo) and trophectoderm (gives rise to placenta). This will assess the concordance of analysis of trophectoderm biopsy to the ‘true’ diagnosis of all the cells in these blastocysts Expected outcomes: Novel findings in assessing human embryos in totality on the karyotypes of all the cells in these abnormal human embryos. This will reflect on the accuracy of current clinical assessment using trophectoderm biopsy in determining the karyotype of human embryos. It will also suggest novel biological phenomena of embryo division and development.	Abnormal embryos identified as aneuploid, will not be used for any fertility treatments. The embryologists will discard these abnormal embryos in accordance with protocols, applicable legislation and associated provisions.	Patients undergoing Assisted Reproductive Therapy (ART)/IVF treatment Embryologists will perform the procedures and biopsy these embryos Scientists from the preimplantation genetic diagnostic laboratory will need to analyse the biopsied cells and generate reports to confirm the karyotype of these cells Clinical coordinators and clinicians would need to follow regulations in counselling and recruiting couples into the study
**Case 3: Discarded oocyte research** Understand the mechanisms and biology of immature oocytes as well as unfertilised oocytes of human origins Principle of study: This is a ‘futuristic’ study whereby examining these immature and unfertilised oocytes from women undergoing ART allows the in-depth understanding of the plausible mechanisms why oocytes fail to develop during ART and fail to fertilise. Expected outcomes: This will allow the discovery of possible causes of failure in oocyte development and ability to fertilise and will identify novel markers for potential therapeutic interventions	Immature oocytes and unfertilised oocytes meant for discarding and never utilised for fertility treatment	Women who underwent ART and need to perform egg pick ups Couple must agree together to allow unfertilised oocytes to be donated for research Embryologists must diagnose immature oocytes and unfertilised oocytes and discard these as per protocols

IVF, in vitro fertilisation.

An important overarching question not considered here due to the focus of this argument, but certainly important to consider in the broader context, is whether couples only producing chromosomally abnormal embryos should be able to successfully request the transfer of an abnormal embryo, such as one with Trisomy 21 or Down syndrome.

We do not include case 4 (see below) in the Table because it is emerging research that warrants a section of its own for more detailed description.

### Test case 1: time lapse imaging and embryo selection

This study involves comparing two different methods of embryo selection: conventional light microscopic examination vs time lapse imaging. Since this involves the selection and transfer of an embryo, it constitutes future person embryo research and raises significant ethical issues. For such research to be justified, equipoise must exist between the two methods of selection: that is, there is genuine uncertainty regarding the superiority of one treatment over another.^25^

A further example of this kind of research is the use of AI on time lapse images to select embryos most likely to implant.^19^ It is essential that such novel interventions are tested prior to widespread acceptance and use—hence, this type of research is ethically required. Although AI embryo selection does involve the transfer of an embryo and the possible creation of a future person, it is philosophically peculiar in one way. It does not involve manipulation of the embryo, rather it involves selecting which embryo is implanted. It is thus an identity-altering intervention: it changes which future person will exist.

As non-destructive observational research, it does not risk harming the future person but rather the fertility patients, who may have an embryo with a lower chance of implantation or perhaps some genetic or chromosomal abnormality if the AI is not functioning as purported. In this way, AI embryo selection is more akin to tissue research representing risks to the couple, rather than embryo research capable of harming a future person. And if equipoise exists (ie, it appears from existing evidence to be at least as good as alternative methods of selection), a randomised controlled trial is ethically appropriate.

However, currently, these new selection procedures can be introduced without clinical trials as a part of clinical judgement. AI embryo selection does not require evidence from randomised controlled trials to be approved as a device by regulatory authorities.^19^ As one of us has argued,^19^ this is unethical and there is an urgent need to approve such research to stop the premature introduction of such selection interventions without proper scientific research and good evidence. Ethics review committees should facilitate such research.

### Test case 2: confirmation of diagnosis of aneuploidy

This is non-future person embryo research; the embryo would be destroyed because it is not clinically suitable for implantation due to its chromosomal abnormality on preimplantation genetic screening, which would render it unsuitable for transfer. For this reason, such processes cannot be considered additional destruction. As the testing procedures involve some costs and risks, it is essential to assess the accuracy of the procedure involved. There is the potential for conflict of interest if the same embryologist making the decision that an embryo is not implantable is also conducting the research. Such a conflict of interest is easily addressed in this context when the decision is based on obvious chromosomal aneuploidy; in these cases, there is little scope for ‘personal judgement’ that might create a conflict (the situation is different if there is possible mosaicism).

### Case 3: discarded oocyte research

This research involves gametes and not embryos. It is ethically similar to non-future person embryo research and low risk. It involves immature eggs which are too undeveloped to be used in forming embryos for transfer to treat infertility. They are ‘excess’ to the needs of the infertile couple. The purpose of this research is to understand the nature of egg development and the capacity to fertilise and create an embryo. This will not involve any transfer of egg or embryo so will not and could not affect any future person. Since this research potentially will generate knowledge that will improve fertility treatment, which has risks in itself, there is an ethical imperative to pursue it. Ethics committees should quickly approve it and encourage participation.

As we have argued, such research is more akin to tissue research because it is studying oocyte development and is not destructive embryo research. Provided informed consent from the cell donors is obtained, there are no obvious reasons why oocyte research should be treated differently than other types of reproductive tissue research that is routinely conducted without special ethical scrutiny. Ethical scrutiny for this type of research is warranted where, for example, a decision will have to be made about whether an egg is too immature for possible fertilisation. This could create a conflict of interest if the embryologist making this decision was both involved in the clinical care and research. Similarly, the IVF clinician will need to make a decision about stimulation of the woman to retrieve eggs for treatment. If the clinician is also engaged in research on immature eggs, this might create a conflict of interest in how hard to stimulate the woman.

Both of these potential conflicts of interests can be addressed by having independent verification of decisions, or blinding clinicians/embryologists as to which patients are participating in the research or by separating clinical care from research, that is, having different embryologists and clinicians involved in the clinical care and research if this is practicable. This may not be physically feasible if it is a small IVF unit where all the staff that is, IVF clinicians and embryologists, have to be on a rota to manage all couples. In this case, convergence of two clinicians may be sufficient to address potential conflict of interest.

### Case 4: a possible project in the future: regeneration of aged oocytes with engineered granulosa/cumulus cells (not listed in table 1)

As a woman ages, her reproductive capacity diminishes due to the decrease in oocyte quantity and quality. To improve reproductive success rates for older women, researchers aim to rejuvenate human oocytes through innovative cellular therapy, by rejuvenating the somatic cells (granulosa/cumulus cells) which surround the maturing oocyte. The mammalian ovarian follicle composed of the oocyte and its surrounding somatic cells—granulosa (including cumulus) and theca cells—is the smallest functional unit to maintain reproductive capacity in women.

The somatic cells, especially granulosa cells (GC), provide the energy, nutrients and microenvironment for the development and growth of the oocyte, allowing the oocyte to undergo maturation to be competent for fertilisation, resulting in a viable embryo. Age-associated senescence or apoptosis (death) of somatic cells leads to atresia (degeneration) of the follicles and is believed to be the basis of age-related decline in oocyte quality, which can be measured via the assessment of key indicators such as spindle morphology, chromosome segregation and mitochondrial distribution. The quality of these markers corelates with the fertilisation potential for the oocyte; poor spindle morphology, chromosome segregation and mitochondrial distribution associated with the lower fertilisation potential of older oocytes, while they exhibit high quality in younger oocytes, with high fertilisation rates. Researchers aim to improve the above markers and rejuvenate the oocyte from aged women though the rejuvenation of the microenvironment of the oocyte via these somatic cells. To prove this feasibility in vitro, they aim to recapitulate the ‘youthful’ ovarian follicle by employing GCs from younger individuals to aid in the growth and maturation of oocytes derived from older individuals to rescue age-related decline in oocyte quality.

In this case, the researchers would create an in vitro chimeric follicle, initially through proof-of-concept in the mouse model. On confirmation of the rescue of age-related decline of oocyte quality through rejuvenation of these old oocytes by GCs from a young individual in the mouse, they would perform pilot human studies using GC cells derived from a young individual (<35 years old), in addition to GC cells and oocytes donated for research from an older woman during IVF (>35 years old). This would involve harvesting GCs and immature oocytes donated for research from young and older women undergoing IVF. Granulosa as well as the cumulus cells are denuded and discarded during IVF, as they are no longer needed for subsequent process of IVF or intracytoplasmic sperm injection.

Once established with primary GC lines derived from young women, researchers would enhance the function of GCs by gene editing them with CRISPR-Cas9 to make them more efficient for aged oocyte rejuvenation. Additionally, these engineered GCs would become innovative cell technology which could be applied to optimise in vitro maturation techniques to enhance the success of maturation but ability to correct age-related decline of oocytes (eg, aneuploidy). The goal of generating engineered GCs would be to use them in vitro, to nurture and rejuvenate poor-quality oocytes. They would be removed prior to IVF in future application, and therefore not enter the human body. The rejuvenated oocyte would then be fertilised and transferred to the woman.

#### Ethical evaluation of case 4

This project aims to manipulate the cells supporting the egg to enhance its development. It does not affect an embryo directly. However, it does potentially modify the egg which produces an embryo, and so raises the higher risks of Future Person Embryo Research if the modified egg is to be used in reproduction.

What are the risks to the future person then? The biological effects expected to be observed from this improved oocyte quality would be a healthy oocyte with normal karyotype and bioenergetics which would enable normal fertilisation. Ideally, this would result in the development of a healthy embryo for implantation and eventually in a healthy live birth. The technology employed is more on the supporting somatic cells to the oocytes, which would be engineered to be ideal ‘rejuvenation machines’ meant to improve and enhance oocyte quality. Therefore, in this case, it is an indirect beneficial effect from these supportive cells to rejuvenate an aged or poor-quality oocyte, already selected for IVF, to ‘healthy’. This is predicated on the fact that the technique described would improve the quality, or fertilisation potential, without genetic changes to the oocyte. Issues relating to the ‘long term’ effects of such a cellular therapy will be difficult to predict in terms of epigenetic changes but current observational longitudinal data on IVF conceived children provides reassuring safety data, as ‘healthy’ oocytes are an indicator of IVF success.

This project appropriately trials the technology first in mice. It would be important to minimise risks, for example, checking whether eggs or embryos so produced are healthy, as qualified by factors such as those named above, before they are transferred and gestated, as to abrogate the need for unnecessary embryo creation or destruction. However, the potential to improve fertilisation is a social and moral good that ethics committees ought to facilitate, and only when it is trialled in humans with the intent to produce a baby should highest level of scrutiny apply due to the possible impacts on the future person.

## The most controversial research: future person affecting research

The most controversial kind of research is that which can change the life course of a future person. An obvious example would be gene editing, for example, to correct major genetic abnormalities such as Tay Sach’s disease or less severe conditions such as thalassaemia major, cystic fibrosis or Huntington’s disease. Most jurisdictions in the world forbid this kind of research but there have been calls for a translational pathway to such trials.^26 27^

To some, it might seem that such research could never be justified. However, for those who will be born with life threatening genetic disorders, it could be literally life-saving.

What seems impossible fast becomes a clinical reality. For example, preimplantation screening is not a standard part of clinical care in many parts of the world. Controversy around the usefulness of preimplantation genetic testing-aneuploidy (A) (PGT-A) for these young female patients remains. However, there is growing evidence that PGT-A improves clinical outcomes in the conditions in which the risk of embryo aneuploidies might increase, such as advanced maternal age, recurrent pregnancy loss, repeated implantation failure, severe male infertility factor.^26–32^ It involves biopsing the trophectoderm from the day 5 embryo (blastocyst) to determine if the embryo has the correct set of chromosomes (euploid) to deem it chromosomally normal for embryo transfer. Years ago, it might have seemed incomprehensible to remove a part of the embryo to check its genetics. Indeed, in some countries it is still considered ‘experimental’ and can only be done under a research protocol. While such research is the most controversial, if sufficient evidence accrues, the benefits could outweigh the risks and it could, in some circumstances, be ethical to proceed.

## Conclusion

There are ethical obligations to conduct research that contributes to generalisable knowledge and improves reproductive health, and this should include embryo research in jurisdictions where it is permitted. Often, the controversial nature of embryo research can alarm ethics committee members, which can unnecessarily delay important research that can potentially improve fertility for patients and society more broadly. Such delay is ethically unjustified. Moreover, countries like the UK, Australia and Singapore have legislation which unnecessarily captures low-risk research such as observational research in an often cumbersome and protracted review process. Such countries should revise such legislation to better facilitate low-risk embryo research.

We have introduced a philosophical distinction to help decision-makers more efficiently identify higher risk embryo research from that which presents no more risks to persons than other types of tissue research. That distinction is between future person embryo research and non-future person embryo research, noting there are caveats in between.

Embryo research is most controversial and deserving of detailed scrutiny when it potentially affects a future person. Where it does not, it should generally require less ethical scrutiny. We have explored a variety of ways in which research can affect a future person, including by deriving information about that person, and manipulating eggs or sperm before an embryo was created.

## Data Availability

Data sharing not applicable as no datasets generated and/or analysed for this study.
